# Tumor‐Associated Lactic Acidosis and Early Death in Patients With Lymphoma

**DOI:** 10.1002/cam4.70824

**Published:** 2025-03-28

**Authors:** Bahaa Atamna, Alon Rozental, Mohammad Haj Yahia, Gilad Itchaki, Ronit Gurion, Moshe Yeshurun, Pia Raanani, Ofir Wolach

**Affiliations:** ^1^ Institute of Hematology, Davidoff Cancer Center Rabin Medical Center Petah‐Tikva Israel; ^2^ Faculty of Medical & Health Sciences Tel Aviv University Tel Aviv Israel; ^3^ Institute of Hematology Meir Medical Center Kfar‐Saba Israel

**Keywords:** early death, lactatemia, lymphoma, Warburg effect

## Abstract

**Background:**

Cancer is characterized by accelerated glycolysis with enhanced glucose uptake and lactate production, a phenomenon termed Warburg effect (WE). We studied the incidence and clinical impact of Warburg‐driven lactic acidosis in lymphoma.

**Methods:**

Patients admitted with newly diagnosed or relapsed/refractory lymphoma and documented lactate levels during the first week of admission were included. Patients with lactatemia were classified as secondary (with a recognizable cause for elevated lactate) or none (WE group).

**Results:**

WE and secondary lactatemia were documented in 58 and 44 patients (15% and 12% of evaluable patients, respectively). Both WE and secondary lactatemia were associated with poor short‐term survival. WE at presentation correlated with tumor burden, with most patients having aggressive disease, advanced stage, and extranodal involvement. WE was associated with high rates of early death (26% and 43% at 30‐ and 60‐days, respectively). Higher lactate levels correlated with worse survival. Earlier initiation of chemotherapy was associated with a (nonsignificant) trend toward better outcomes, whereas steroid and/or thiamine therapy did not alter patient outcomes. Glucose administration was associated with worse survival.

**Conclusion:**

WE‐driven lactatemia is associated with high tumor burden and increased short‐term mortality in lymphoma. Prompt initiation of anti‐lymphoma therapy may improve outcomes.

## 1 Introduction

Lactic acidosis is a medical emergency that reflects, in most cases, tissue depletion of oxygen supply due to hypoperfusion, such as in septic shock (type A lactic acidosis). Conversely, type B lactic acidosis occurs under normoxic conditions (without evidence of organ hypoperfusion) and is usually a result of drug or toxin disruption of cellular metabolism or from nutritional deficiency states, such as in thiamine deficiency [1, 2, 3]. Type B lactic acidosis has also rarely been reported in association with solid and hematological malignancies (the Warburg effect [WE]) [4, 5]. The mechanism underlying this association is incompletely understood and may be partly explained by enhanced aerobic glycolytic activity in the cancer cell triggered by oncogenic lesions, such as the expression of hypoxia‐inducible factor‐1α (HIF‐1α). These changes ultimately promote increased glucose uptake by the tumor and diversion from the normal oxidative process toward a glycolytic pathway with the production of lactate [5].

This metabolic alteration grants cancer cells important advantages, including (1) the ability to adaptat to different oxygen concentrations, (2) the production of avid glycolytic intermediates that can be further efficiently utilized as metabolic substrates for nucleotide, fatty acid, and amino acid biosynthesis pathways, and (3) the evasion of potential cellular oxidative damage that is typically a sequela of oxidative phosphorylation secondary to aerobic cellular respiration [6]. Furthermore, lactate can act as an oncometabolite that binds the GPR81 receptor and activates the PI3K–Akt–mTOR pathway, and contributes to HIF‐1 stabilization, promoting malignant progression [7, 8, 9, 10]. Extracellular acidosis is another detrimental trait arising from aerobic (and anaerobic) glycolysis. Its key role in driving malignant progression and resistance to conventional therapies has been previously reviewed [11, 12, 13].

The clinical implications of elevated lactate levels and lactic acidosis that result from WE are not well‐studied in patients with malignant processes. Few case reports and series demonstrated a possible correlation between early death, disease aggressiveness and elevated lactate in sera of patients that presented with hematologic malignancies [14, 15, 16].

In this study, we systematically evaluate the prognostic and clinical implications of lactatemia resulting from WE as well as from other etiologies and assess the impact of different interventions on disease course and early mortality.

## 2 Methods

### 2.1 Patients

A retrospective study of consecutive patients admitted to the Haemato‐Oncology department in a large tertiary medical center between the years 2013–2022 was conducted. Patients with documented lactate levels during the first week of admission were analyzed. Patients with hematological diseases other than lymphoma were excluded. Medical records of patients with elevated lactate were reviewed by two physicians (B.A. and O.W.) for a recognizable cause for elevated lactate, or none (assigned as the WE group). In our institution, lactate levels are reported as a part of a rapid and comprehensive oximetric/metabolic panel. Lactate results are reported along with oximetrics, hemoglobin levels, acid–base status (pH, bicarbonate, pco2, po2, etc.), glucose levels, and electrolytes, and these tests were ordered as indicated for relevant patients. The upper limit of normal (ULN) lactate level is 19 mg/dL according to our local laboratory.

For each patient, we retrieved demographics, underlying hematologic disease, comorbidities, laboratory results, and therapeutic interventions. For patients with multiple lactate tests during the first week of admission, maximal lactate levels were documented. The date of death was retrieved from medical records, and survival was calculated from the first day of index admission. Early death (ED) was defined as death from any cause within 30 days of admission. This study was approved by the Institutional Review Board (IRB) of the Rabin Medical Center (reference code 0829‐22‐RMC); written informed consent was exempt by the IRB due to the retrospective design of this study.

### 2.2 Statistical Analysis

Descriptive statistics were used for baseline characteristics. Categorical data are presented using frequencies and percentages, and continuous variables are described with medians and ranges. Overall survival (OS) was defined as the interval time from the day of index admission to either the time of death from any reason or the last recorded follow‐up date. OS rates for both 1 and 5 years were computed for the entire cohort and specifically for patients with lactate levels above 25 mg/dL, a cutoff chosen since it was shown to effectively separate clinical outcomes among tested patients in an exploratory analysis of receiver operating curves at different lactate thresholds. Kaplan–Meier survival analysis was employed to compare observed median OS with lactate levels above 25 mg/dL and IV dextrose, supplemented by Log‐rank test assessments. Univariate analyses were performed by Cox proportional hazard regression, and a *p* value of < 0.05 was considered statistically significant. All of the data were analyzed by using IBM SPSS Statistics version 26.0.

## 3 Results

### 3.1 Patients

**FIGURE 1:**
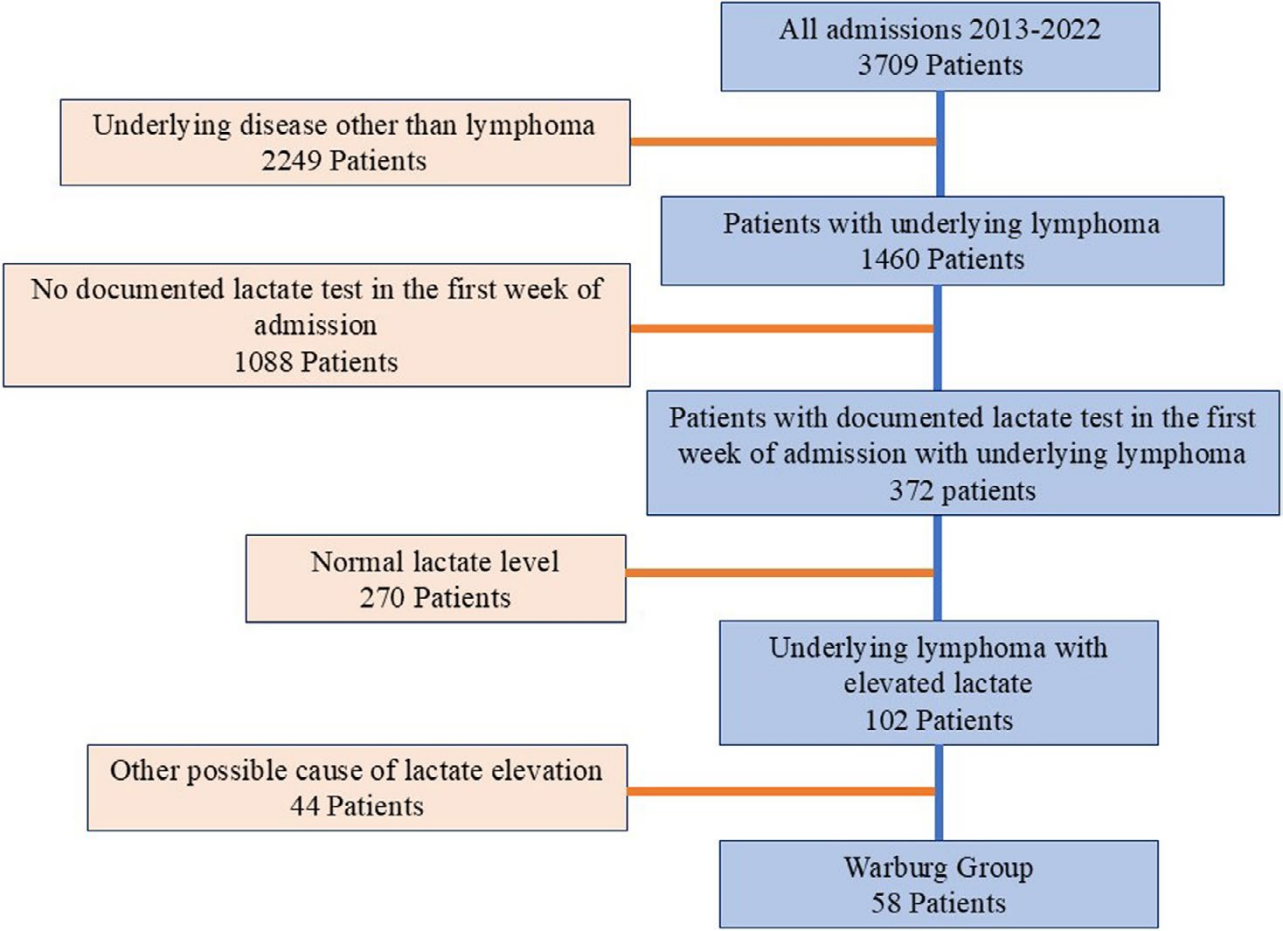
CONSORT diagram.

Of 3709 consecutive admissions between January 2013 and December 2022, 1460 patients were admitted with newly diagnosed or relapsed/refractory (R/R) lymphoma, and 372 had a documented lactate test in the first week of admission. One hundred and two patients had lymphoma and elevated lactate (lactate levels ≥ 20 mg/dL); 58 patients fulfilled the criteria for WE, representing an incidence of 15% of all lymphoma admissions with available lactate levels. Forty‐four patients had their high lactate level attributed to causes other than WE (12% of all lymphoma admissions with available lactate levels) including infectious (*n* = 31) and noninfectious causes (concomitant metformin use *n* = 9 or other *n* = 4) (CONSORT diagram, Figure 1).

Median age of patients with WE was 63 years (range 25–86) and 60% (*n* = 35) were males. Seventy‐two percent of patients had aggressive lymphoma. Diffuse large B‐cell lymphoma was the most frequent type of diagnosis (*n* = 33) followed by T‐cell Lymphoma (*n* = 8) and Hodgkin's lymphoma (*n* = 6). Most patients (60%) had newly diagnosed lymphoma during their study index admission, whereas the remainder had R/R disease. Most patients had advanced disease, with stage III–IV in 95% of patients, and 81% had extranodal involvement of their disease. The median maximal lactate level was 31.5 mg/dL (range 20–114; ULN −19 mg/dL; Table S1). The median levels of LDH, total bilirubin, aspartate aminotransferase (AST) and alanine aminotransferase (ALT) were 942 U/L (range 324–1081, ULN 480), 1 mg/dL (0.11–13.5, ULN 1.2), 35 U/L (10–531, ULN 31) and 27 U/L (8–613, ULN 34), respectively. Three patients had grade 3–4 elevations in total bilirubin associated with grade 3 elevations of ALT or AST. These patients had widespread involvement of their lymphoma with suspected liver involvement. Only one patient had documented hypoglycemia (Table S1).

Corticosteroids were administered to 55 patients with WE (94.8%); 13 patients (22.4%) were treated with thiamine; and 12 patients (20.7%) were given IV glucose supplements (Table S2).

### 3.2 Outcomes

**FIGURE 2:**
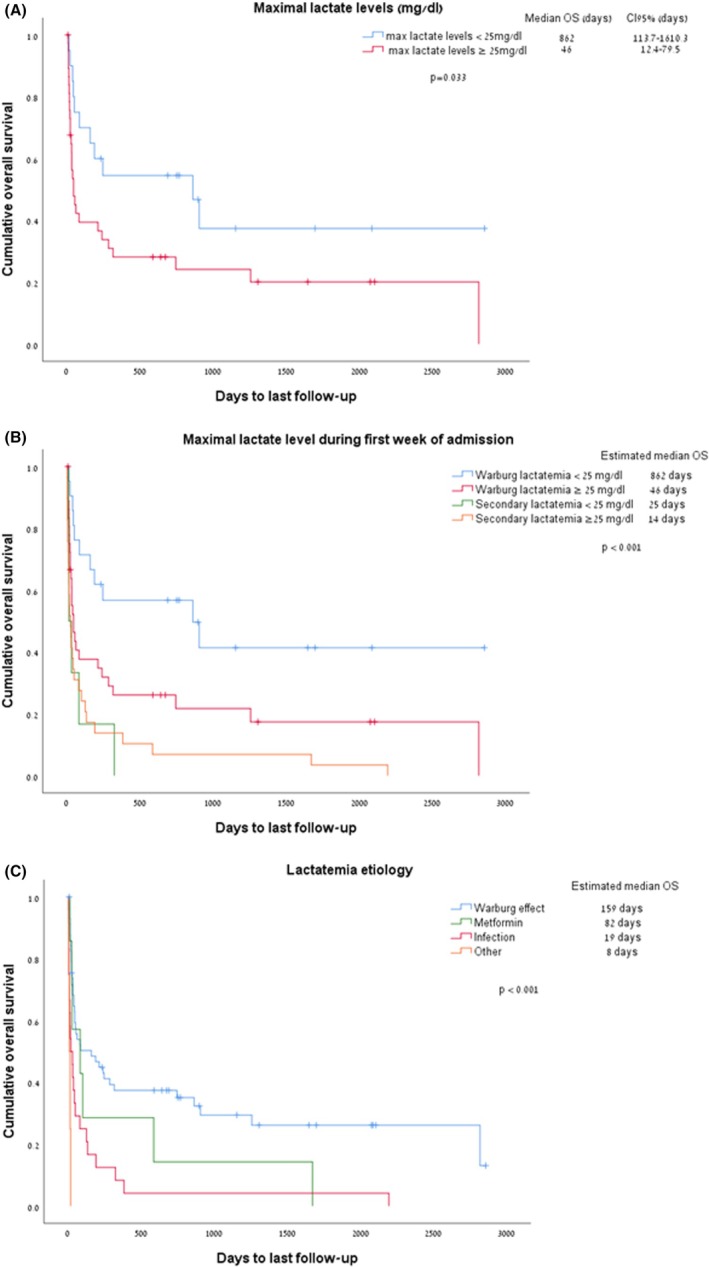
(A) Kaplan–Meier analysis for survival in patients with WE with lactate levels ≥ 20 but < 25 mg/dL (blue curve), and lactate levels of ≥ 25 mg/dL (red curve). (B) Survival rates of patients analyzed by Warburg effect vs. secondary lactatemia, and level of lactate. (C) Kaplan–Meier analysis for survival in patients with different causes of lactatemia.

Over one‐third of patients with WE (36%, *n* = 21) died during the index admission. ED (within 30 days) was documented in 26% (*n* = 15) of patients, and 60‐day mortality was 43%. Subgroup analysis of patients with higher lactate (lactate ≥ 25 mg/dL) showed higher rates of early death (47% died during the index admission, 36% within 30 days; Table 1). In most patients, the cause of death was lymphoma‐related (*n* = 21) followed by infections (subsequent events unrelated to the initial lactatemia, *n* = 13; Table S3). Higher lactate levels were associated with worse survival, with a median survival of 46 days for those with lactate levels ≥ 25 mg/dL, compared to 862 days for patients with above‐normal lactate but lower than 25 mg/dL (*p* = 0.03; Figure 2A).

**TABLE 1:** Early mortality in patients with WE.

	Lactate ≥ 20 mg/dL	Lactate ≥ 25 mg/dL
Death during index admission, *n* (%)	21 (36.2%)	18 (47.4%)
Mortality 30 days (ED), *n* (%)	15 (25.9%)	13 (36.1%)
Mortality 60 days, *n* (%)	25 (43.1%)	20 (55.6%)

When considering all causes of elevated lactate (*n* = 102), secondary lactatemia (*n* = 58) portended worse survival rates than in WE patients (*n* = 42) (median OS of 25 vs. 159 days, *p* < 0.001; Figure S1), with higher lactate levels associated with worse outcomes. Lactatemia secondary to hemorrhagic shock and hypoxemia (labeled as “others” in Figure 2C) and sepsis carried the worst survival rates of 8 and 19 days, respectively (*p* < 0.001, Figure 2B,C).

Earlier initiation of anti‐lymphoma directed therapy (chemotherapy, immunotherapy, or both) was associated with a nonsignificant trend toward a better outcome; chemotherapy was initiated at an average of 5.5 days after documentation of the first elevated lactate in patients who survived at least 30 days (range (−)4–30), versus 9.05 days after the first elevated lactate in patients with ED (range 0–25, *p* = 0.13).

**FIGURE 3:**
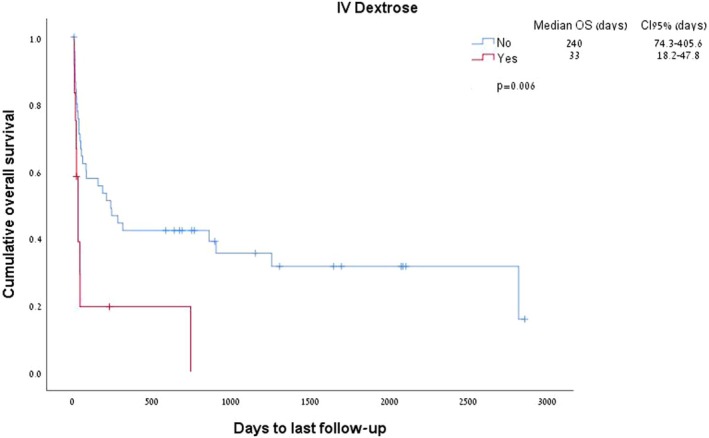
Dextrose administration and survival of patients with the Warburg effect.

Both corticosteroids administration and thiamine therapy were not associated with better survival (HR 1.682 [95% CI 0.8–3.5], *p* = 0.166 for thiamine, and HR 1.598 [95% CI 0.38–6.6], *p* = 0.52 for steroids). Interestingly, dextrose administration (*n* = 12, 20.6% of patients) was associated with reduced survival (*p* = 0.006, Figure 3).

**FIGURE 4:**
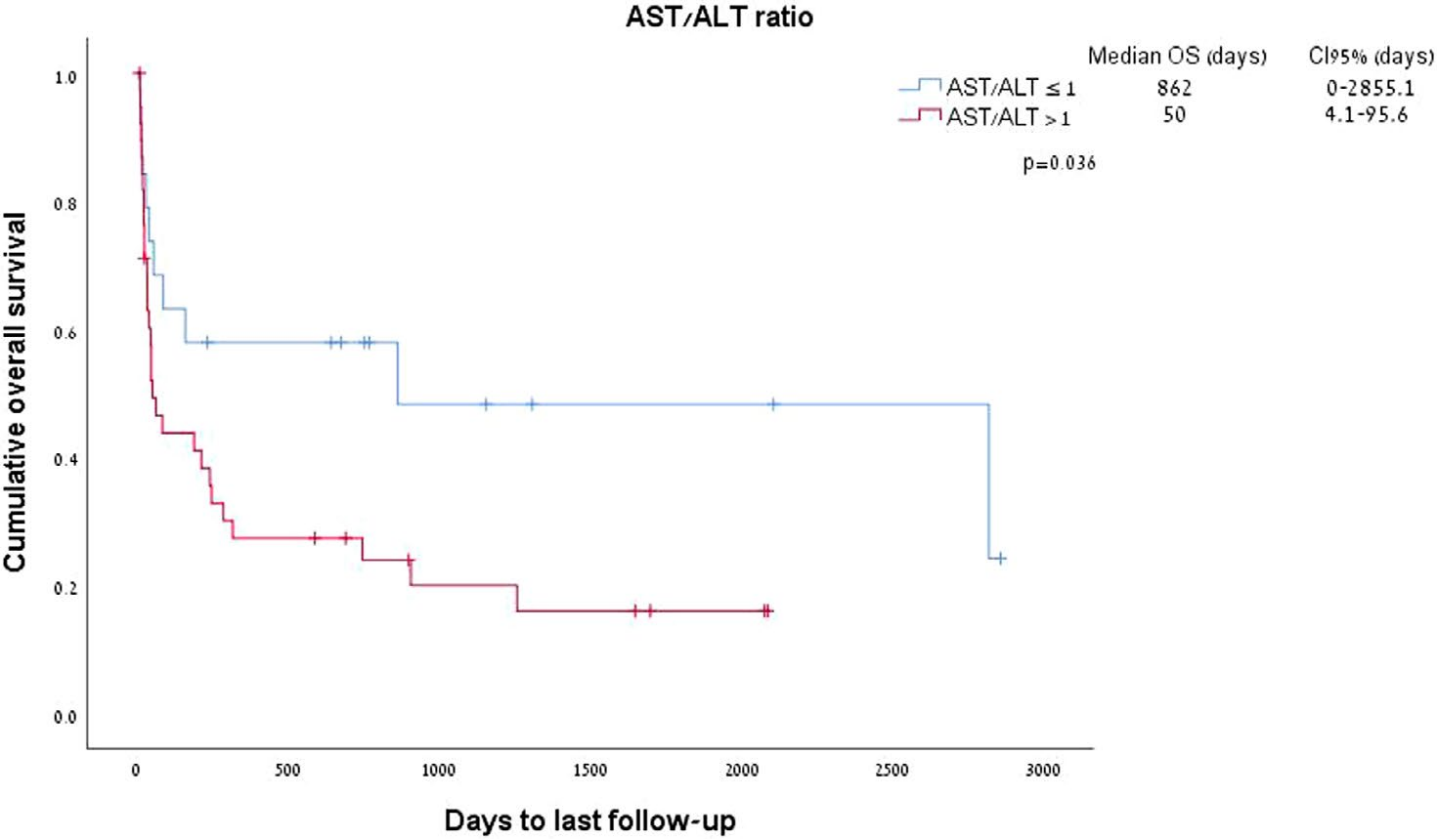
AST:ALT ratio (above vs. below 1) and survival of patients with Warburg effect.

The ratio between AST and ALT emerged as a significant predictor of survival in patients with WE. An AST:ALT ratio > 1 was documented in 67% of patients with WE and was associated with a median survival of 50 days as compared to 862 days in patients with an ALT ratio of < 1 (*p* = 0.03; Figure 4).

## 4 Discussion

WE was first described by Dr. Warburg in 1923 [17, 18] and is well characterized in cancer on the cellular level. This metabolic phenomenon results in accelerated glycolysis despite aerobic conditions producing large amounts of lactate. A systemic rise in lactate and lactic acidosis has been reported in hematological malignancies and reflects both increased production of lactate secondary to high tumor burden and impaired lactate turnover or elimination by the liver or kidney.

In this study, we conduct a systematic analysis to evaluate the clinical impact of lactate levels in patients admitted with lymphoma. We demonstrate that among lymphoma in‐patients with elevated lactate, type B lactic acidosis attributed to WE is not rare (15% of assessed patients) and confers very poor short‐term survival. Patients with lactatemia attributed to WE carry a grim prognosis, with 30‐ and 60‐day mortality occurring in 26% and 43%. Higher levels of lactate correlate with poor survival rates, implying a high tumor burden and more aggressive disease.

Our results are consistent with previous reports that demonstrated a correlation between ED and elevated lactate in the sera of patients with hematological malignancies (Table 2).

**TABLE 2:** Warburg effect in lymphoma and early death: literature review.

Publication	*N*	Clinical context	Early death
**Current publication**	**58**	**Retrospective review of admissions to a large hematology department**	**26%**
Chaba et al. [16]	46	Retrospective review of admissions to a large intensive care unit	NR
Sillos et al. [15]	3	Case series of pediatric T‐ALL lymphoma/leukemia	66%
	27	Literature review for cases until 2001	74%
Friedenberg et al. [14]	4	Retrospective review of patients between 1990 and 2003 in single center	50%
	8	Literature review for cases between 1990 and 2006	100%
Di Comite et al. [19]	1	Case report. Gastric diffuse large B‐cell NHL	Yes
Glasheen et al. [20]	1	Case report. Burkitt's lymphoma	Yes
He et al. [21]	1	Case report. Relapsed NK/T‐cell lymphoma	Yes
Deenadayalan et al. [22]	1	Case report. Intravascular DLBCL	Yes

*Note:* The bold row represents the data from the current study.

Friedenberg et al. published in 2007 a retrospective review of patients with hematological malignancies between 1990 and 2003 in a single center. Of the 7 patients reported with type B lactic acidosis and hematologic malignancy, 4 had lymphoma, 2 were diagnosed with CLL, and one patient had AML; patients demonstrated poor short‐term survival, including 3 patients that died within 1 week of admission [14].

Sillos et al. published a literature review of 25 patients with lymphoma and type B lactic acidosis and showed a 74% ED rate for these patients. Of note, the threshold of lactate assigned in the two above‐mentioned studies was 5 meq/L (~45 mg/dL) as compared to a threshold of only 20 mg/dL in our study, supporting our observation that higher mortality rates correlate with higher lactate levels (Figure 2A and Table 1) [15].

Chaba et al. analyzed the effect of elevated lactate on survival in patients with lymphoproliferative diseases admitted to a large intensive care unit. Patients with WE (*n* = 46) and elevated lactate without WE (*n* = 20) had a 70% mortality rate at 1 year as compared to 38% in patients without elevated lactate levels. Factors associated with WE in this analysis included bone marrow involvement, hypoglycemia, concomitant tumor‐lysis syndrome, and a high sequential organ failure assessment score (SOFA score) [16].

Several interventions have been proposed for this acute and life‐threatening condition. Thiamine administration did not improve outcomes in our analysis, nor did earlier steroid administration. Earlier administration of anti‐lymphoma therapy (chemotherapy or immunotherapy) trended with better survival outcomes but failed to reach statistical significance. These data imply that elevated lactate is a marker of more aggressive disease and may play a role in disease resistance, as proposed earlier [7, 23]. We also demonstrate that intravenous glucose administration was associated with poor survival. Although this observation may merely reflect a selection of acutely ill patients with high tumor burden for this therapy, it may also suggest a potentially aggravating effect for glucose in this clinical scenario.

The observation of reduced survival in patients with the Warburg group treated with intravenous glucose, as seen in our series, was previously described, in one report of a patient with a bulky undifferentiated carcinoma. The administration of hypertonic glucose induced lactic acidosis by increasing the availability of glucose, which is the limiting factor of aerobic glycolysis, and thus increasing the production of lactate by the tumor [24]. Sillos et al. attempted to treat two patients with type B malignancy‐associated lactic acidosis with insulin and glucose in an attempt to increase the conversion of pyruvate to acetyl‐coenzyme A, and consequently facilitate the oxidation of lactate to pyruvate. However, this intervention produced no appreciable improvement in the acidosis, and was suggested to increase lactate production [15].

AST to ALT ratio, also termed the De Ritis ratio, was shown to correlate with survival in our analysis. We initially sought to assess the AST/ALT ratio in the context of WE based on our clinical observations, and since previous reports suggest that these enzymes may correlate with metabolic stress and systemic organ damage [25]. The AST/ALT ratio was first proposed in the study of hepatitis [26] and is commonly used to differentiate between causes of liver disease. Additional studies showed that this metabolic biomarker can be used as an effective prognostic biomarker for non‐liver diseases, such as cardiovascular disease [27], and in various cancers [25, 28, 29, 30].

In our analysis, secondary lactatemia (related to causes other than WE) correlated with higher mortality and poor outcomes. The majority of such patients had a hypotensive crisis and subsequent lactatemia (type A lactic acidosis), reflecting critical deterioration and impending multiorgan failure.

This study is limited by its retrospective design and the potential for selection bias as lactate values at admission were available for only 1259 of 3709 screened patients. This potentially represents a selected patient population with a more acute clinical presentation and may overestimate the actual incidence of WE in lymphoma patients. Nonetheless, our analysis represents a large systematic review of lactatemia and WE in patients with lymphoma, and is the first to test different interventions and their clinical impact on outcomes. Future prospective observations are needed to validate our findings. Whether tumor‐associated lactatemia is a pathogenic driver of resistance and poor outcome in these patients, or only a biomarker for tumor burden and aggressiveness is not clear.

In conclusion, this study highlights the acute presentation and poor outcome of tumor‐associated lactatemia and defines prognostic metabolic markers to be used in the assessment and management of these patients.

This high‐risk clinical scenario is likely underrecognized and therefore underdiagnosed. Clinicians should be aware of this condition. Early lactate monitoring is suggested, and prompt initiation of anti‐lymphoma therapy should be considered in cases of WE.

## Author Contributions

Study design: Bahaa Atamna, Ofir Wolach. Data collection and analysis: Bahaa Atamna, Alon Rozental, Mohammad Haj Yahia, Gilad Itchaki, Ronit Gurion, Moshe Yeshurun, Pia Raanani, Ofir Wolach. Bahaa Atamna, Alon Rozental, and Ofir Wolach wrote the manuscript. All authors critically revised the manuscript.

## Ethics Statement

This study was approved by the Institutional Review Board of the Rabin Medical Center.

## Consent

The authors have nothing to report.

## Conflicts of Interest

The authors declare no conflicts of interest.

## Supporting information


Tables S1–S3

## Data Availability

For original data, please contact owolach@gmail.com.
