# Supplementary material for: Tumor‐Associated Lactic Acidosis and Early Death in Patients With Lymphoma
Source: Cancer Med. 2025 Mar 28;14(7):e70824. doi: 10.1002/cam4.70824 (PMC11951173; doi:10.1002/cam4.70824)
Supplement: Supplementary file 1 — Tables S1–S3 [file CAM4-14-e70824-s001.docx]

**Table S1A: Patients’ characteristics in WE group n=58.**

| 63 (range25-86) | | **Age, years (median, range)** |
| --- | --- | --- |
| Female 23 (39.7%) | Male 35 (60.3%) | **Gender (n, %)** |
| 23 (39.7%) | DLBCL | **Diagnosis (n, %)** |
| 6 (10.3%) | DLBCL transformed from low grade lymphoma |  |
| 3 (5.2%) | High grade T cell lymphoma |  |
| 3 (5.2%) | Mantle cell lymphoma |  |
| 2 (3.4%) | Follicular lymphoma |  |
| 2 (3.4%) | PTLD (DLBCL) |  |
| 6 (10.3%) | Hodgkin’s lymphoma |  |
| 1 (1.7%) | Burkitt's lymphoma |  |
| 2 (3.4%) | Anaplastic large T cell lymphoma |  |
| 2 (3.4%) | Intravascular DLBCL lymphoma |  |
| 2 (3.4%) | Prmiary mediastinal large B cell lymphoma |  |
| 2 (3.4%) | Peripheral T cell lymphoma |  |
| 1 (1.7%) | Sezary syndrome |  |
| 1 (1.7%) | Primary cutaneous T cell lymphoma |  |
| 1 (1.7%) | SLL |  |
| 1 (1.7%) | Marginal zone lymphoma |  |
| 0 (0%) | 1 | **Lymphoma stage (n, %)** |
| 3 (5.2%) | 2 |  |
| 8 (13.8%) | 3 |  |
| 47 (81%) | 4 |  |
| 35 (60.3%) | Newly diagnosed | **Disease status**  **(n, %)** |
| 23 (39.7%) | Relapsed/refractory |  |
| 0 (0%) | 0 | **ECOG**  **(n, %)** |
| 17 (29.3%) | 1 |  |
| 21 (36.2%) | 2 |  |
| 20 (34.5%) | 3 |  |
| 31.5 (20-114) | | **Maximal lactate levels*, mg/dL (median, range)** |
| 942 (324-1081) | | **LDH***  **U/L (median, range)** |
| 35 (10-531) | | **AST***  **U/L (median, range)** |
| 27 (8-613) | | **ALT***  **U/L (median, range)** |
| 1 (0.11-13.5) | | **Bilirubin (total)***  **mg/dL (median, range)** |

*Upper limit of normal for the following values: lactate 20 mg/dL, LDH 480 U/L, AST 31 U/L, ALT 34 U/L, Bilirubin (total) 1.2 mg/L. LDH -lactate  dehydrogenase, AST - Aspartate Aminotransferase, ALT - Alanine transaminase.

**Table S1B – Patients’ characteristics in secondary lactatemia group n=44.**

| Median 65 (range 25-88) | | **Age years (median, range)** |
| --- | --- | --- |
| Female 18 (41%) | Male 26 (59%) | **Gender (n, %)** |
| 15 (34.1%) | DLBCL | **Diagnosis (n, %)** |
| 2 (4.5%) | DLBCL transformed from low grade lymphoma |  |
| 3 (6.8%) | Mantle cell lymphoma |  |
| 4 (9.1%) | Follicular lymphoma |  |
| 1 (2.2%) | PTLD (DLBCL) |  |
| 2 (4.5%) | Hodgkin’s lymphoma |  |
| 5 (11.4%) | Burkitt's lymphoma |  |
| 6 (13.6%) | Anaplastic large T cell lymphoma |  |
| 4 (9.1%) | Peripheral T cell lymphoma |  |
| 2 (4.5%) | Marginal zone lymphoma |  |
| 0 (0%) | 1 | **Lymphoma stage (n, %)** |
| 3 (6.8%) | 2 |  |
| 8 (18.2%) | 3 |  |
| 33 (75%) | 4 |  |
| 24 (54.5%) | Newly diagnosed | **Disease status (n, %)** |
| 20 (45.5%) | Relapsed/refractory |  |
| 0 (0%) | 0 | **ECOG (n, %)** |
| 4 (9.1%) | 1 |  |
| 23 (52.3%) | 2 |  |
| 17 (38.6%) | 3 |  |
| 20-207 (median 39.5) | | **Maximal lactate levels (mg/dL, range)** |

**Table S2: Supportive interventions (Patients, %)**

| Steroids | 55 (94.8%) |
| --- | --- |
| Thiamine | 13 (22.4%) |
| Dextrose | 12 (20.7%) |

| **Table S3: Cause of death in WE group through follow up period**   \| Infectious \| 13 (22.4%) \| \| --- \| --- \| \| Disease related \| 21 (36.2%) \| \| Other \| 5 (8.6%) \| \| Total \| 39 (67.2%) \|   Figure S1  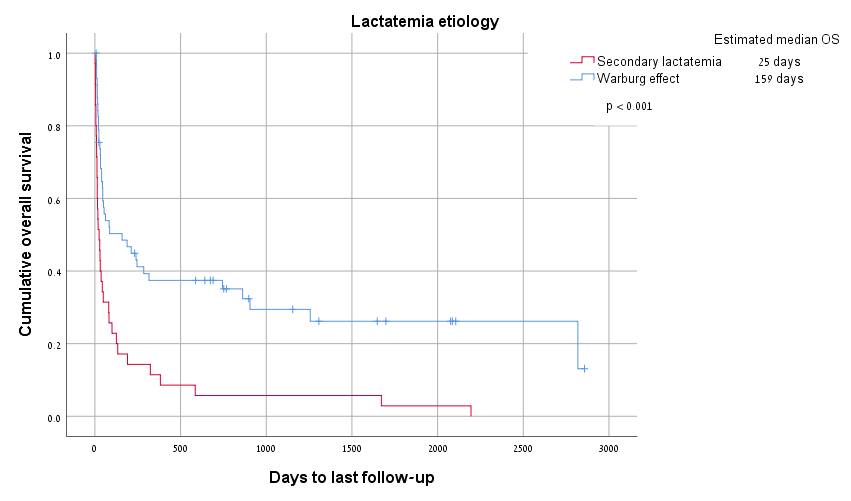  Figure S1: Survival rates of patients with lactatemia attributed to WE, and lactatemia secondary to other causes than WE. |
| --- | --- | --- | --- | --- | --- | --- | --- | --- |
